# Taxol Crystals Can Masquerade as Stabilized Microtubules

**DOI:** 10.1371/journal.pone.0001476

**Published:** 2008-01-23

**Authors:** Margit Foss, Buck W. L. Wilcox, G. Bradley Alsop, Dahong Zhang

**Affiliations:** 1 Department of Zoology, Oregon State University, Corvallis, Oregon, United States of America; 2 Molecular and Cellular Biology Program, Oregon State University, Corvallis, Oregon, United States of America; 3 Center for Genome Research and Biocomputing, Oregon State University, Corvallis, Oregon, United States of America; Duke University Medical Centre, United States of America

## Abstract

Taxol is a potent anti-mitotic drug used in chemotherapy, angioplastic stents, and cell biology research. By binding and stabilizing microtubules, Taxol inhibits their dynamics, crucial for cell division, motility, and survival. The drug has also been reported to induce formation of asters and bundles composed of stabilized microtubules. Surprisingly, at commonly used concentrations, Taxol forms crystals that rapidly bind fluorescent tubulin subunits, generating structures with an uncanny resemblance to microtubule asters and bundles. Kinetic and topological considerations suggest that tubulin subunits, rather than microtubules, bind the crystals. This sequestration of tubulin from the subunit pool would be expected to shift the equilibrium of free to polymerized tubulin to disfavor assembly. Our results imply that some previously reported Taxol-induced asters or bundles could include or be composed of tubulin-decorated Taxol crystals. Thus, reevaluation of certain morphological, chemical, and physical properties of Taxol-treated microtubules may be necessary. Moreover, our findings suggest a novel mechanism for chemotherapy-induced cytotoxicity in non-dividing cells, with far-reaching medical implications.

## Introduction

In 1971, Taxol (paclitaxel) was isolated from the Pacific yew tree, *Taxus brevifolia*, and shown experimentally to have antitumor activity [1]. Through work in the laboratory of S. B. Horwitz, Taxol's novel mechanism of action was discovered: it binds to and stabilizes microtubules, inhibiting progression through the cell cycle [2]. More specifically, Taxol stabilizes microtubules [3] by binding stoichiometrically (or substoichiometrically [4]) within their lumen [5]. The consequent loss of microtubule dynamics is thought to impair the mitotic spindle, thus causing cell cycle arrest at the metaphase-to-anaphase transition, and ultimately, cell death by apoptosis [2], [6].

Taxol has been used extensively as an antitumor drug (reviewed in [7]), and more recently in drug eluting stents to prevent reblockage of coronary arteries after balloon angioplasty (reviewed in [8]). In addition to its medical applications, Taxol is frequently used in research for studying microtubules and microtubule-based structures, such as cilia, flagella, spindles, asters, and bundles. It has also been widely reported to induce extensive polymerization of microtubules, and their assembly into bundles and/or asters (e.g., [4], [9]–[15]), particularly at higher concentrations.

In this study we have documented an unexpected property of the drug that could necessitate a reinterpretation of many microtubule-based studies, and that has the potential for major medical implications. Specifically, Taxol forms crystals that superficially resemble microtubules, and that sequester the tubulin building blocks of microtubules. In other words, crystallized Taxol bound by free tubulin can masquerade as polymerized tubulin bound by free Taxol.

## Results and Discussion

**Figure 1:**
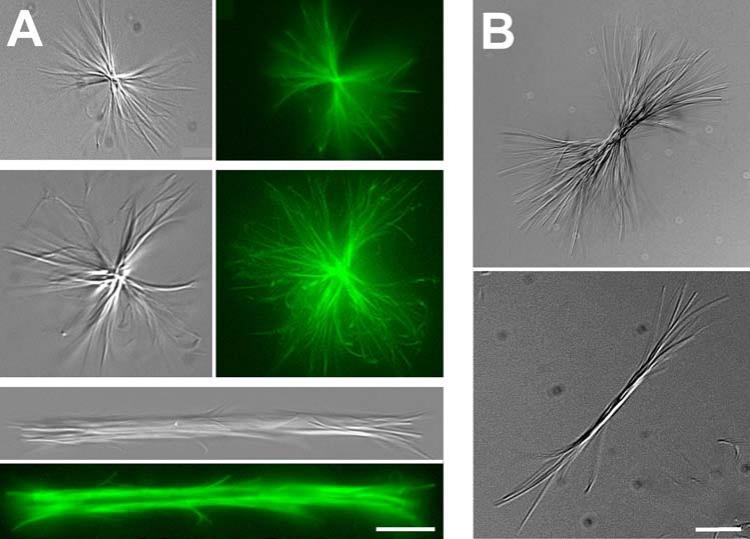
Taxol crystals resemble asters and bundles formed in the presence of Taxol-stabilized microtubules. (A) Two asters (top and middle) and one bundle (bottom) formed in the presence of Taxol and fluorescently labeled tubulin, as in text. Paired images with DIC (left) and fluorescent (right) optics. (B) DIC images of a Taxol crystal aster (top) and bundle (bottom), formed in the absence of tubulin. Bars, 10 µm.

Taxol is known to function by binding the inner surface of microtubules and stabilizing them in their polymerized state [2], [5]. Thus, we expected to observe only individual microtubules in a commonly used microtubule assembly buffer containing fluorescently labeled tubulin and 20 µM Taxol (as in Materials and methods). Unexpectedly, asters and bundles were also observed (Fig. 1 A), despite the absence of cellular constituents thought to be required for their formation (e.g., [16]–[18]). These structures could be seen both by fluorescence and differential interference contrast (DIC) microscopy. More surprisingly, asters and bundles were also present in an otherwise identical control solution lacking tubulin, and could be visualized only by DIC microscopy (Figs. 1 B, 3 B).

Given the low solubility of Taxol in aqueous solutions [19], and its tendency to form needle-like dihydrate crystals [20], we deduced that we were seeing Taxol crystals. Apparently, the crystalline needles had self-assembled into bundles and asters that closely resembled the Taxol-stabilized microtubule structures reported in many studies (e.g., [9], [21]). Assembly could have occurred by some combination of collision of needles in solution, lateral nucleation on preexisting needles, or nucleation of multiple needles on a single impurity (speck of dust etc.). We hypothesized that Taxol crystals could bind fluorescent tubulin subunits, allowing the decorated crystals to masquerade as stabilized microtubule structures (Fig. 1 A).

**Figure 2:**
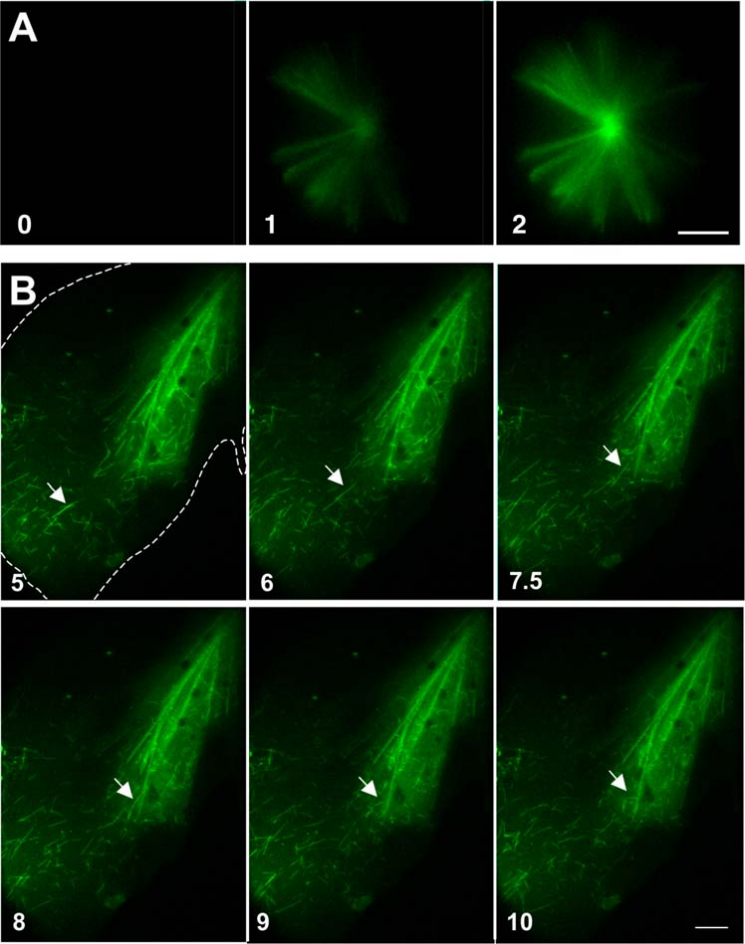
Taxol crystals bind fluorescently labeled tubulin subunits. (A) Fluorescently labeled tubulin (released from a micropipette to the left of the field) immediately accumulated on the preformed Taxol crystal aster, as shown in these sequential images. (B) An equilibrium solution of fluorescently labeled tubulin (green haze) and microtubules was released as in (A), and decorated a Taxol ‘bow-tie’ (i.e., nonspherical) crystal aster (half of aster is visible, in upper right). Again, the crystal was instantly labeled by subunits, which diffuse more rapidly than microtubules. Microtubules did not appear to contribute to the brightness of the tubulin-decorated crystal, as fluorescence intensity did not increase over time. Thus, microtubules did not appear to play a major role in decorating the Taxol crystal. The arrow tracks the path of a single microtubule that briefly made contact with the aster, but diffused away. The shallow aqueous puddle containing the crystal aster is outlined in the first panel. Time in seconds. Bars, 10 µm.

To test our hypothesis, we prepared Taxol crystals, and exposed them to fluorescently labeled tubulin as follows. Stock Taxol in solvent was diluted in buffer to 20 µM, creating crystalline asters and bundles. The crystals were pipetted under oil onto a chamber slide. Ice-cold, fluorescently labeled tubulin subunits were micropipetted into the chamber, to one side of the crystals, allowing us to visualize their trajectory in real time using fluorescence microscopy. The crystals began to fluoresce almost instantly (Fig. 2 A), consistent with rapid binding of tubulin to the crystal rather than the slower assembly of microtubules on the crystal surface. In a separate experiment, tubulin was allowed to assemble into microtubules and the equilibrium solution of microtubules and tubulin subunits was pipetted near the crystal. Once again, the crystals were labeled almost instantly by the subunits, whereas the slower-moving microtubules were observed to diffuse past the crystal (Fig. 2 B). Both experiments suggested that crystals were decorated by free tubulin rather than by microtubules. Furthermore, since free Taxol binds the inner surface of microtubules, it would be topologically impossible for intact microtubules to bind the crystal via the previously characterized Taxol-binding site [5].

**Figure 3:**
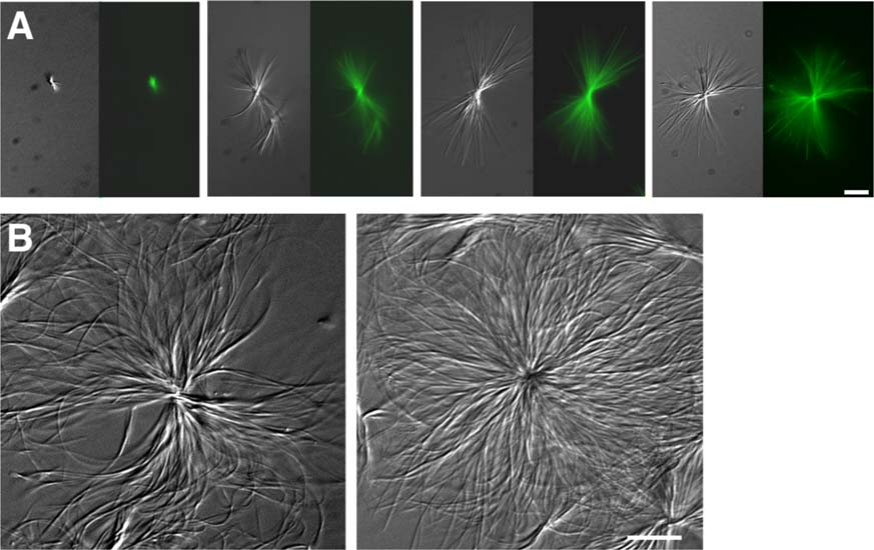
Concentration- and time-dependence of aster formation. (A) Fluorescently labeled Taxol-induced asters (DIC and fluorescent optics) formed in polymerization solutions stabilized with a concentration gradient of Taxol (left to right): 0.92 µM, 1.0 µM, 10 µM, 20 µM. Asters were not visible at or below 0.88 µM Taxol. These results are consistent with the low solubility of Taxol in aqueous solution [19]. (B) Taxol crystals, formed in 20 µM Taxol in aqueous buffer. Asters were visualized by DIC microscopy 24 hours after preparation. Compare to the smaller, less-dense aster in Fig. 1 B, which was visualized within 10–60 minutes. Crystals were not a solvent-induced artifact, as no structures were visible in a control solution lacking Taxol. Bars, 10 µm.

The formation of tubulin-decorated crystal asters and bundles was dependent on Taxol concentration (Fig. 3 A). Asters were not observed when microtubules were stabilized with concentrations below ∼0.9 µM. At Taxol concentrations above ∼0.9 µM, asters formed in 15 minutes or less (Fig. 3 A). Crystallization occurred progressively faster as the concentration increased. Asters formed even during preparation of 1–20 µM Taxol, if dilutions from stock Taxol were not made quickly and mixed thoroughly. Furthermore, longer incubations of saturated Taxol solutions (compare Fig. 1 B to Fig. 3 B), as well as higher concentrations of Taxol (compare Fig. 3 A, 1 µM vs 20 µM Taxol), produced asters and bundles with longer needles.

Taxol has often been used under conditions in which crystallization is certain or likely to occur (based on issues raised in [19], [22]). There is an incongruity between the low solubility of Taxol in aqueous solution and the considerably higher concentrations at which the drug is often used. Taxol is soluble in Dimethyl sulfoxide solvent, but upon dilution into aqueous solutions at concentrations greater than 0.77 µM (0.66 µg/mL), it eventually precipitates [19]. Thus, conclusions based on studies using concentrations in the micromolar range may be subject to misinterpretation. Even at low nanomolar concentrations in the extracellular medium, Taxol can be concentrated by cultured cells, which can readily attain intracellular saturation (i.e., [Taxol]>0.77 µM) [22]. Rate of uptake differs from one cell line to the next and must be determined experimentally [23]. Significantly, intracellular Taxol concentrations are rarely measured.

Our results imply that some previously reported Taxol-induced asters or bundles could either include or be composed of tubulin-decorated Taxol crystals, and could easily be mistaken for Taxol-stabilized microtubules. Similarly, a single decorated crystalline needle could resemble an individual stabilized microtubule. Thus, it would be prudent to reevaluate morphological, chemical, and physical properties of Taxol-treated microtubules, such as sedimentation, turbidity, rigidity, birefringence, affinity, stability, and thermodynamics of assembly. Of course, we do not mean to suggest that all or most previous work on Taxol is suspect or invalid. We are merely stating that in certain cases, particularly those involving high concentrations of Taxol, or those resulting in unexpected or contradictory conclusions, it is worth considering an alternative explanation.

For example, VanBuren et al. summarize published values for flexural rigidity of the microtubule, as measured by various methods, with and without Taxol stabilization [24]. There is an astounding range of values. We hypothesize that some disagreement may stem from the inadvertent use of tubulin-decorated Taxol crystals in studies in which microtubules were stabilized with saturated concentrations of Taxol.

In addition, our data are inconsistent with reports that high concentrations of Taxol can induce polymerization of massive quantities of microtubules, in vivo or in vitro (see, for example, [9], [4]). Our results imply that Taxol crystals would sequester tubulin from the subunit pool, shifting the equilibrium of free to polymerized tubulin in a direction disfavoring assembly. Taxol-induced polymer content in cells has been measured by isolating cytoskeletons in order to separate microtubules from free tubulin. However, because tubulin-decorated Taxol crystals would be expected to co-sediment with the cytoskeleton, centrifugation-based assays could overestimate the amount of microtubule-stabilized polymer by disregarding the presence of decorated crystals. Turbidity-based assays could result in similar overestimates.

In contrast, very low, subsaturated concentrations of Taxol would indeed be expected to induce additional polymerization by stabilizing existing microtubules, shifting the equilibrium toward polymerization. This was demonstrated by the study of Derry et al., in which they determined, in vitro, the effects of increasing concentrations of Taxol on the mass of microtubule polymer [4]. The mass was reported to increase in a biphasic manner, with an increase over controls for 10–50 nM Taxol (by polymerization), no significant increase between 50 and 500 nM Taxol, and a larger increase between 1 µM and 20 µM. The latter increase could be due to the presence of tubulin-decorated Taxol crystals rather than newly polymerized Taxol-stabilized microtubules. We have cited just two observations in the literature that could be explained by the presence of decorated Taxol crystals. However, we hope that our work may provide an opportunity for others to reevaluate previous research, resolve additional anomalies, and increase awareness of Taxol's strengths and weaknesses as a research tool.

More importantly, should Taxol crystallize in living cells due to its progressive uptake [22], there could be far-reaching medical implications. Cytotoxicity in non-dividing cells could result from the presence of crystals and their sequestration of free tubulin, rather than by Taxol's direct interference with the mitotic spindle. For example, peripheral neuropathy is a debilitating side effect of Taxol chemotherapy [25]. Neurons, which heavily utilize microtubule-dependent vesicle transport, may be especially vulnerable to the presence of Taxol crystals. Our hypothesis is supported by the discovery that Taxol-treated cultured bovine aortic endothelial cells are defective in microtubule-dependent vesicle transport [26]. Again, intracellular crystallization could provide a mechanism by which transport is impaired.

Neutropenia (characterized by abnormally low neutrophil counts) is another serious side effect of Taxol chemotherapy [27]. Neutrophils and platelets are derived from a common myeloid progenitor, and both circulate in the blood. Electron micrographs of Taxol-exposed chilled platelets demonstrate a striking reorganization of microtubules: from the usual circumferential bands to radiating bundles and “sheaves of wheat”-like structures [28]. Their appearance indicates that Taxol may crystallize within platelets. It could be interesting to examine the microtubule configuration in Taxol-treated neutrophils.

Furthermore, in a clinical study, Jiko et al. measured post-chemotherapy Taxol concentrations in blood serum, revealing a positive correlation between duration of levels greater than 0.1 µM and percent decrease in platelet number. Indeed, serum levels remained above 1 µg/ml (1.2 µM) for approximately four hours [29]. (Neutrophils are similarly affected by high concentrations of Taxol [29].) Neutrophils and platelets in Taxol-containing serum are in some ways analogous to cultured cells in Taxol-containing medium. Caov-3 ovarian carcinoma cells attain a high intracellular Taxol concentration (10.7 µM) after four hours in medium containing 30 nM Taxol (and 99.7 µM after 24 hours in 1 µM Taxol) [22]. Thus, it is not unreasonable to think that blood cells may also attain saturation at clinically applicable concentrations, potentially resulting in neutropenia or other hematological aberrations.

Notably, pharmacological efficacy tended to correlate negatively with high ‘maximum Taxol concentration’, leading Jiko et al. to conclude that the dosage in their study may be excessive [29]. This observation substantiates the well-established mechanism by which low concentrations of Taxol [30] are sufficient to disrupt cell division, by stabilizing spindle microtubules. It is also consistent with our proposed mechanism by which somewhat higher (but still clinically relevant) concentrations are cytotoxic.

Given that Taxol may crystallize intracellularly, it could be worth determining whether or how other anti-mitotic drugs crystallize intracellularly or form non-microtubule polymers that may be decorated by tubulin. For example, colchicine has been reported to induce a variety of tubulin-containing arrays that start out as transitory cortical strands. With prolonged exposure to colchicine, they transform into “needle-type bundles, arranged as different crystalloids and/or macrotubules” [31]. Discodermolide has been reported to increase the microtubule polymer mass and induce bundling of microtubules [32], and laulimalide enhances microtubule polymerization, based on turbidity [33]. We hope our data will provide a steppingstone for others to determine whether Taxol or similar drugs can crystallize intracellularly, and if so, under what conditions.

## Materials and Methods

### Microscopy

For micropipetting experiments, a custom-made slide with an ∼75 mm^3 ^chamber was used. For each treatment, a 0.75 µl sample was pipetted into the chamber under hydrophobic Halocarbon 400 oil to prevent evaporation. Otherwise, each 0.75 µl sample was sandwiched between a glass slide and a cover slip, and sealed with silicone grease. The small sample volume results in flatter droplets, thus improving image clarity. Samples were visualized at room temperature using a digital-enhanced DIC microscope (Zeiss Axiovert-100) equipped with a 1.4 NA/63X Plan-Apochromat objective and a 1.4 NA achromatic-aplanatic condenser. Images were captured with a CCD digital camera (MicroMax, Princeton Instruments, Inc.), and processed using Image Pro Plus (Media Cybernetics) and Adobe Photoshop.

### Preparation of Taxol crystals

A stock solution of 1 mM Taxol in Dimethyl sulfoxide (DMSO) was diluted to 20 µM in BRB 80 buffer (80 mM PIPES, 1 mM EGTA, 2.1 mM MgCl_2_, 1 mM GTP, 4% glycerol) [34] in a microcentrifuge tube. Crystals were removed from the tube and pipetted under Halocarbon 400 oil into a chamber slide. We confirmed that the crystals were actually present in larger volumes of solution (e.g., 100 µL) in the microcentrifuge tubes, and were not merely forming upon contact with the glass slide. Using both plastic tips and glass micropipettes, we pipetted crystals from tube to slide while observing the transfer under the microscope. The crystals emerged from the tips preformed, and could be seen immediately at multiple focal planes, not just at the focal plane adjacent to the glass.

### Generation of Taxol-stabilized microtubules

To promote microtubule assembly, 1.14 mg/ml Fluorescein-tubulin (Cytoskeleton) in BRB 80 buffer was incubated at 37°C for 20 minutes [34]. Stabilization buffer, consisting of 20 µM Taxol (Molecular Probes) in BRB 80, was added to the microtubule polymerization reaction. For concentration-dependence of aster formation, 0.25 µM to 20 µM Taxol was tested (Fig. 2).

### Crystal micromanipulation and decoration

Micropipettes were pulled from glass capillaries (World Precision Instruments) using a Flaming/Brown micropipette puller (Sutter Instruments), and maneuvered with a Burleigh MIS-5000 series piezoelectric micromanipulator. For decoration of the crystals with tubulin, 2.5 mg/ml ice-cold fluorescently-labeled tubulin in BRB 80 was back-loaded into a micropipette (tip diameter ∼0.1 µm) using a chilled 2 µl Hamilton syringe. A Narishige microinjector was used to control tubulin flow. For decoration of the crystals with an equilibrium mixture of tubulin and microtubules in BRB 80, a micropipette of tip diameter ∼0.3 µm was used. The tip opening was wide enough that intact microtubules could pass through (as seen in Figure 2 B).
